# Increased cardiac contractility by decreased HAX-1 expression

**DOI:** 10.1186/1479-5876-10-S2-A65

**Published:** 2012-10-17

**Authors:** Wen Zhao

**Affiliations:** 1 School of Pharmaceutical Sciences, Zhengzhou University, 100 Kexue Avenue, Zhengzhou, Henan 450001, China

## Background

The HS-1 associated protein X-1 (HAX-1) is a ubiquitously expressed protein that protects cardiomyocytes from programmed cell death. HAX-1 is mainly located in cardiac mitochondria and sarcoplasmic reticulum. Recently, it has been recognized that HAX-1 serves as a binding partner of phospholamban, which plays a fundamental role in controlling basal contractility and constitutes a key downstream effector of the β-adrenergic signaling cascade. However, the functional significance of HAX-1 in the heart remains unclear. Our previous studies have shown that overexpression of HAX-1 *in vitro* or *in vivo* by adenoviruses and transgenesis reduced cardiac myocyte contractility and calcium transients under basal condition without significant alterations of isoproterenol response. Conversely, *in vitro* downregulation of HAX-1 enhanced calcium kinetics and mechanics under basal conditions.

## Methods and results

**Figure 1:**
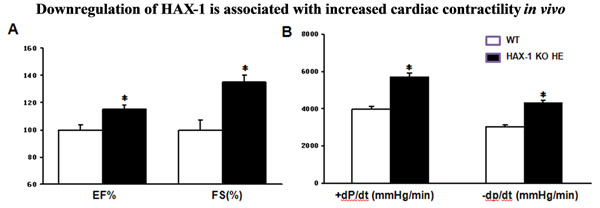
WT: Wild type, HAX-1 KO HE: HAX-1 knockout heterozygous. A: EF% (percentage of ejection fraction) and FS% (percentage of fractional shortening) by echocardiography; B: Rates of contraction (+dp/dt) and relaxation (-dp/dt) by Langendorff perfusion. *: P<0.05, compared with WT.

To further investigate the role of the endogenous HAX-1 in the cardiac contractile function, HAX-1 heterozygous deficient mice with 36% of HAX-1 expression in the heart were characterized, since the homozygous mice are lethal at 5-12 weeks afterbirth. Interestingly, *in vivo* echocardiography showed that decreased HAX-1 expression was associated with significantly enhanced cardiac performance, including fractional shortening and ejection fraction, when compared to age-matched wild types. *Ex-vivo* Langendorff perfusion suggested markedly increased rates of contraction and relaxation, compared to wild types (Figure 1). Furthermore, at the cardiomyocyte levels, we also found similar cardiac phenotype with elevated fractional shortening, rates of contraction and relaxation as well as calcium kinetics under basal conditions. The functional improvement in the heterozygous HAX-1 deficient mouse hearts does not plays a role in the expressions of major SR calcium handling proteins, including: SERCA2a, calsequestrin and phospholamban. However, the affinity of SERCA2 for calcium was significantly increased without alteration of maximal velocity of this calcium pump. The enhanced cardiac contractility is not related to any significant cardiac remodeling and histology changes at the age of 10-12 weeks.

## Conclusion

These results indicate that decreased HAX-1 expression in the heart is associated with increased cardiac contractility and calcium handling, suggesting that HAX-1 may be a novel regulator in cardiac contractile performance.

